# Automatic assessment of time-resolved OCT images for selective retina therapy

**DOI:** 10.1007/s11548-016-1383-6

**Published:** 2016-04-11

**Authors:** Sarah Zbinden, Şerife Seda Kucur, Patrick Steiner, Sebastian Wolf, Raphael Sznitman

**Affiliations:** ARTORG Research Center Biomedical Engineering Research, University of Bern, Murtenstrasse 50, 3008 Bern, Switzerland; Department of Ophthalmology, Inselspital Bern, Freiburgstrasse 12, 3010 Bern, Switzerland

**Keywords:** Computer-assisted intervention, Retinal laser therapy, Time-resolved OCT, Treatment outcome estimation, Feature design

## Abstract

**Purpose:**

In recent years, selective retina laser treatment (SRT), a sub-threshold therapy method, avoids widespread damage to all retinal layers by targeting only a few. While these methods facilitate faster healing, their lack of visual feedback during treatment represents a considerable shortcoming as induced lesions remain invisible with conventional imaging and make clinical use challenging. To overcome this, we present a new strategy to provide location-specific and contact-free automatic feedback of SRT laser applications.

**Methods:**

We leverage time-resolved optical coherence tomography (OCT) to provide informative feedback to clinicians on outcomes of location-specific treatment. By coupling an OCT system to SRT treatment laser, we visualize structural changes in the retinal layers as they occur via time-resolved depth images. We then propose a novel strategy for automatic assessment of such time-resolved OCT images. To achieve this, we introduce novel image features for this task that when combined with standard machine learning classifiers yield excellent treatment outcome classification capabilities.

**Results:**

Our approach was evaluated on both ex vivo porcine eyes and human patients in a clinical setting, yielding performances above 95 % accuracy for predicting patient treatment outcomes. In addition, we show that accurate outcomes for human patients can be estimated even when our method is trained using only ex vivo porcine data.

**Conclusion:**

The proposed technique presents a much needed strategy toward noninvasive, safe, reliable, and repeatable SRT applications. These results are encouraging for the broader use of new treatment options for neovascularization-based retinal pathologies.

## Introduction

**Fig. 1:**
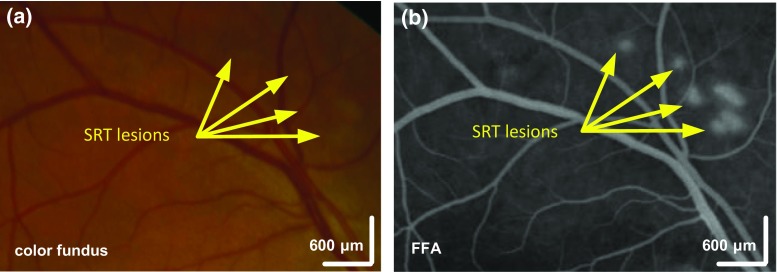
SRT lesions in **a** color fundus image and **b** fundus fluorescein *angiography* (FFA) for the same eye region. With appropriate laser energies, lesions after selective retina therapy remain invisible in color fundus image while being visible in FFA (state of the art for the identification of lesions introduced by SRT)

Selective retina therapy (SRT) is an efficient laser treatment for a variety of retinal pathologies. At its core, the laser induces a photo-mechanic disruption by evaporating water in targeted cells. In contrast to conventional laser photocoagulation which is prone to induce laser damage to all retinal layers, SRT achieves therapeutic effects by solely targeting the retinal pigment epithelium (RPE) which is responsible for nourishing light absorption cells. By doing so, SRT not only reduces negative side effects of traditional photocaogulation but also facilitates healing of neighboring tissue [3, 5]. In particular, recent studies have shown promising long-term efficacy of SRT in both animal and patient studies [2, 5, 6, 11] when the treatment is executed with laser energies that produce visible lesions in angiography but invisible to ophthalmoscopic imaging, as illustrated in Fig. 1.

While highly beneficial, SRT suffers from the main drawback of missing direct visual feedback regarding the success of the therapy. Introduced lesions remain invisible in conventional ophthalmoscopic imaging due to the absence of coagulation in the inner retinal layers (see Fig. 1). For this reason, the selection of an appropriate laser energy and a reliable monitoring of the therapy are both challenging and critical. The patient-specific concentration of melanin in the RPE [1] further aggravates the determination of the necessary treatment energy level as it influences the rate of conversion from laser energy to heat. For these reasons, real-time and objective evaluation of the introduced tissue damage as it occurs is paramount for safe, reliable, and repeatable SRT.

Current approaches to SRT monitoring are either based on the invasive and time-consuming fundus fluorescein angiography (FFA) [4] or the detection of ultrasonic pressure waves of collapsed cells [14] or change analysis in the backreflection during the presence of laser-induced microbubbles [15]. While the latter methods have already been implemented [12], these approaches suffer from the lack of depth information. Recent research has shown however that optical coherence tomography (OCT) [8], acquired simultaneously with the laser therapy may provide the missing spatial and temporal information necessary for a comprehensive, repeatable, and reliable lesion assessment [16]. In a pilot study, the application of single SRT laser pulses to induce RPE lesions was detectable in OCT data and appeared to correlate well with the extent of tissue damage imaged with FFA. This visible *FFA leakage* is a consequence of the accumulation of contrast agent in sub-retinal tissue which breached the blood–retina barrier. Yet, no method to date has been able to automatically assess laser treatment outcome at specifically targeted locations. This inability severely hinders the clinicians’ capacity to use SRT as a treatment option.

**Fig. 2:**
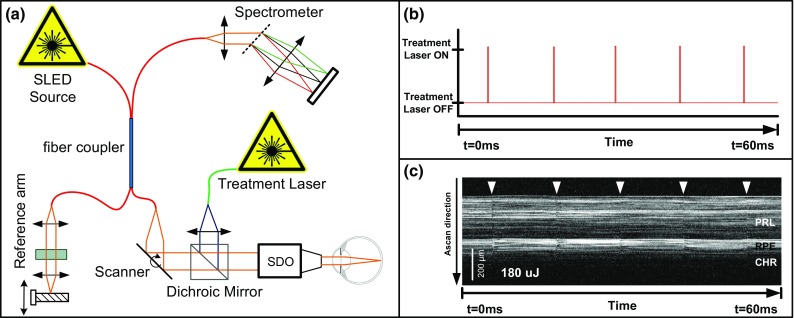
Schematic setup of the combined SRT-OCT system **a** including the SRT treatment system and the measurement and treatment beam being combined using a dichroic mirror. Figures to the *right* show the time sequence of the application of five laser pulses **b** and the corresponding OCT M-Scan data **c** with visible temporal signal variations marked with *white arrows*

To overcome this limitation, this paper introduces the first automatic and observer-independent classification scheme for time-resolved OCT data of SRT lesions. To this end, we introduce novel image features for time-resolved OCT that when combined with traditional classification schemes provide excellent identification of positive and negative treatment outcomes. The proposed approach was evaluated on both ex vivo porcine eye samples and human patients undergoing SRT. In addition, we demonstrate here that our features are reliable for classification, to the point that classification models trained on ex vivo porcine data can effectively be used for prediction in human patients. As such, we show here that the use of time-resolved OCT during SRT can provide a direct feedback to the ophthalmologist and allows SRT to be executed using strongly reduced pulse energies without the risk of undertreatment.

The remainder of the paper is organized as follows: We begin by describing our laser and imaging system in “System overview” section. “Problem statement” section then formalizes our classification problem. “M-Scan features” section describes the proposed approach including the details about the features we propose. “Experimental results” section presents the implementation details and the experimental results of the proposed method. Lastly, “Conclusion” section discusses our findings and concludes the paper.

## System overview

In this section, we describe our SRT-OCT system used for patient treatment. The OCT system used for data acquisition shown in Fig. 2a features a line scan frequency of 70 kHz and a spectral bandwidth of 170 nm centered at 830 nm (EBS8C10, Exalos AG, Switzerland), leading to an axial resolution of 1.78 $\upmu$m in air and is described in detail elsewhere [17]. While a Fourier-domain OCT system was used for the experiments in this paper, any type of OCT system (e.g., time domain, swept source) could be used instead, as long as the system parameters are properly set. The OCT was combined with the treatment system using a dichroic mirror to enable simultaneous data acquisition, and M-Scans (i.e., repeated A-Scans at the same XY position over time) were acquired during each SRT pulse application. Prior to any further analysis and processing, the raw OCT data undergo conventional preprocessing steps such as $\lambda -k$ mapping, numerical dispersion correction, background subtraction, mapping to a color map, and axial motion correction.

The SRT pulses are executed using a frequency-doubled Nd:YLF laser [10] (SRT Vario, Medical Laser Center Lübeck, Lübeck, Germany) with pulse width of 250 ns and a pulse repetition rate of 100 Hz for 30 pulse trains (see Fig. 2b). Laser energy was guided onto the retina using a scanning digital ophthalmoscope (SDO, Wild medTec, Austria), and SRT lesions were placed onto the retina by the ophthalmologist using a built-in manual deflection mirror.

**Fig. 3:**
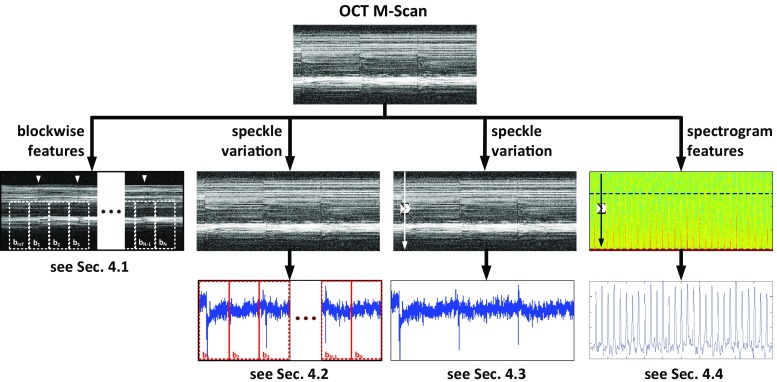
Overview of the proposed features. These include blockwise analysis of M-Scans, speckle variance features, and spectrogram features. See text for details on how these are computed

OCT M-Scans are acquired as a single depth profile at a fixed position on the sample. As such, M-Scans provide a time-resolved depth profile of light scattering and reflection in the tissue under investigation (see Fig. 2c). The temporal resolution thereby depends on the maximal A-Scan rate of the OCT system, and the axial resolution is defined by the coherence length of the employed OCT system. With this, M-Scans are capable of mapping even sub-resolution changes in the scattering structure by abstract variations in the phase of the OCT signal. For SRT, where the laser pulse parameters circumvent thermal effects, the damage in the RPE cells is based on photomechanical rather than photothermal effects [2, 11]. Thus, detectable features in OCT M-Scans may primarily be based on the presence of microbubbles in the RPE cells due to the induced water evaporation. Extensive recent studies have confirmed the presence of a set of distinctive signal variations in the recorded OCT data that correlate well with lesion visibility in fundus fluorescein angiography [16]. It is thus assumed that the presence of temporal OCT signal variations as shown in Fig. 2c represents successful RPE cell rupture.

## Problem statement

In this paper, we consider the problem of automatically identifying the successful (i.e., the presence of RPE cell rupture) and unsuccessful SRT laser application from the corresponding M-Scan image data. Let $X_k$, $k = 1,2,\ldots ,K$, be an $L{\times }T$ M-Scan-OCT image of the *k*th observed SRT laser application with *L* and *T* representing the spatial and temporal dimensions, respectively. Then, let $y_k \in \{0,1\}$ be the label associated with the corresponding $X_k$, where

1 $$\begin{aligned} y_k = {\left\{ \begin{array}{ll} 1 &{} \text {if therapy is successful},\\ 0 &{} \text {otherwise}. \end{array}\right. } \end{aligned}$$

We define a feature extractor function $g :R^{L{\times }T} \rightarrow R^d$ which maps an $L{\times }T$ image $X_k$ to a *d*-dimensional feature vector. Our goal then was to learn a classifier $f :R^{d} \rightarrow \{0,1\}$ assigning a binary label to a given *d*-dimensional vector. In our case, a random forest classifier [7] is used to represent our function *f*. We now introduce a novel feature set *g* to tackle this classification problem.

## M-Scan features

In this section, we describe our approach to the classification problem presented in “Problem statement” section. Accordingly, we describe how representative features can be extracted from M-Scans (see “System overview” section). As shown in Fig. 2c, successful SRT application becomes visible in M-Scans as high-frequency intensity variations in standard OCT images during and following the single SRT pulse application. The physical principle of OCT imaging only allows the detection of the reflectivity and the position of reflective components in the sample tissue. As such, detectable features are limited to variations in intensity, speckle pattern, and the phase of the OCT signal. Features were thus computed from pixel intensity-based speckle images, blockwise image analysis as well as from time–frequency analysis (TFA) using a short-time Fourier transform (STFT). Figure 3 shows an overview of the features we compute here and which we now discuss in detail.

### Blockwise M-Scan features

To represent variations in the temporal pixel intensity distribution of the OCT M-Scan, a blockwise speckle analysis is performed by dividing the OCT M-Scan into equal-size blocks of signal. The blocks are defined such that only one of the two subsequent blocks will contain the temporal position of laser application as shown in Fig. 3.

Let us denote the *n*th block as $B^k_{n}{\in }R^{L_b{\times }T_b}$, $n =1,\ldots ,N-1$, and the reference block $B^k_{0}{\in }R^{L_b{\times }T_b}$ which are taken from the *k*th M-Scan $X_k$. Here, $T_b$ and $L_b$ show temporal and spatial dimensions of each block, respectively. Note that $B^k_{0}$ corresponds to image data before any laser application has been performed. Henceforth, we drop the subscript/superscript *k* from the notation for the sake of simplicity. As the signal variations inside a defined block can be inferred from the variance of the pixel distributions, the following $(N-1)$-dimensional feature vector $u_{1}^{bm}$ is computed as in the following:

2 $$\begin{aligned} u_{1}^{bm}(n) = \sqrt{\frac{1}{\left( L_b T_b - 1\right) }\sum _l^{L_b}\sum _t^{T_b}{\left( B^{'}_n(l,t) - \mu ^{'}_n\right) ^2}}, \end{aligned}$$

where $B^{'}_n = B_n - B_{0}$ is the reference-subtracted block and $\mu ^{'}_{n}= \frac{1}{L_bT_b}\sum _{l,t}^{L_b, T_b}B^{'}_n(l,t )$ is its mean. Moreover, the maximum gradient of the resulting vector $u_{2}^{bm} = \max \left( {\nabla }u_{1}^{bm}\right)$ is also used as a feature. The blockwise M-Scan feature vector then contains the two extracted feature components, i.e., ${u^{bm}}^\intercal = [{u_{1}^{bm}}^\intercal , {u_{2}^{bm}}]$ of size *N*.

### Blockwise speckle features

While large movements alter the spatial intensity distribution in the OCT images, smaller effects may only be detectable in the speckle pattern which, in contrast to the common intensity image representation, is sensitive to sub-wavelength vibrations [13, 18]. In order to provide a broad set of features for classification, time-resolved variations in speckle pattern were calculated from the OCT M-Scans.

To further emphasize signal blocks containing high signal variations, the root mean square (rms) is first computed individually for all temporal sampling points leading to a vector with length *T*. Subsequently, the variance of the vector values within each signal block is computed for all *N* blocks, i.e.,

3 $$\begin{aligned} v_n = \frac{1}{\left( T_b - 1\right) }\sum _{t=1}^{T_b}{\left( R_n(t) - \frac{1}{T_b} \sum _{t'=1}^{T_b} R_n(t')\right) ^2} \end{aligned}$$

where $R_n(t)= \sqrt{\frac{1}{L_b}\sum _{l=1}^{L_b}{{\left| B_n(l,t)\right| }^2}}$ is the rms of *n*th block at temporal position *t* and $v_n$ corresponds to the variance of such rms values within the *n*th block computed along time axis. Then, an $(N-1)$-dimensional blockwise speckle feature vector is extracted by normalizing the computed rms variance vector as in the following:

4 $$\begin{aligned} u^{bs}_1(n) = v_n - v_{0}, \quad \text {for}\quad n = 1,\ldots ,N-1, \end{aligned}$$

where $v_{0}$ holds for the rms variance of the reference block. In addition, we include the maximum value of the resulting vector, that is, $u^{bs}_2 = \max {\left( u^{bs}_1 \right) }$. Accordingly, one gets a blockwise speckle feature vector ${u^{bs}}^\intercal = \left[ {u^{bs}_1}^\intercal , u^{bs}_2\right]$ of size *N*.

### Speckle variance features

A second set of features based on OCT speckle information is computed based on the temporal variation in the speckle values in the OCT signal. For this, we identify the variations along time by the following sum:

5 $$\begin{aligned} S(t) = \sum _{l=1}^{L}{X(l,t)}, \end{aligned}$$

where *S*(*t*) represents the intensity sum for each time step *t* for the OCT image *X*. In order to eliminate the bias due to the varying background, the offset term $S_{\text {off}}(t)$ for the corresponding observation is subtracted in the following:

6 $$\begin{aligned} {S(t)}' = \bar{S}(t) - \bar{S}_{\text {off}}(t), \end{aligned}$$

in which $\bar{S}(t)$ and $\bar{S}_{\text {off}}(t)$ represent zero-mean and unit variance correspondences of *S*(*t*) and ${S_{\text {off}}(t)}$, respectively. The offset term above is computed by the moving average operation. In the resulting vector ${S(t)}'$, time-resolved variations can be quantified with the number of values surpassing an empirical threshold $s_{th}$ as shown by:

7 $$\begin{aligned} u^{sv} = \sum _{t=1}^T{\mathbbm {1}_{\{s \in R |s > s_{th}\}}}\left( {S(t)}' \right) , \end{aligned}$$

where $\mathbbm {1}$ is the indicator function and $u^{sv}$ is the resulting feature related to the speckle variance.

### Spectrogram features

It is believed that the presence of microbubbles in the RPE layer leads to rapid rearrangement of the scattering structure of the retinal tissue which is represented as high-frequency variations in the phase and intensity distribution of the OCT signal. We therefore introduce a time–frequency analysis by means of a short-time Fourier transform (STFT) to encode such signal variations in the feature set.

Here, STFT is performed on the rms signal *R*(*t*) of an OCT sample *X* as

8 $$\begin{aligned} Z(\tau , \omega ) = \sum _{t =1}^{T} R(t)h(t-\tau )\exp {(-j{\omega }t)} \end{aligned}$$

with *h*(*t*) and $Z(\tau , \omega )$ being the windowing function and the STFT of rms signal, respectively. We then perform a median filtering operation on the amplitude of the resulting STFT and call it $Z_m(\tau ,\omega )$. Subsequently, the sum of the median filtered spectrogram magnitudes is found for each time component, i.e.,

9 $$\begin{aligned} \bar{Z}(\tau ) = \sum _{\omega }\left( Z_m(\tau ,\omega )\right) _{dB}, \end{aligned}$$

where $(\cdot )_{dB}$ subscript represents the conversion to the dB scale. With the normalization given by:

10 $$\begin{aligned} \hat{Z}(\tau ) = \frac{\bar{Z}(\tau ) - \min \left( \bar{Z}(\tau )\right) }{ \max \left( \bar{Z}(\tau )- \min \left( \bar{Z}(\tau ) \right) \right) }, \end{aligned}$$

we compute the crest factor [9] in the following

11 $$\begin{aligned} u^{sp}_{1} = \frac{\max \left( \hat{Z}(\tau )\right) }{\sqrt{\frac{1}{{\mathrm{T}}} \sum _{\tau =1}^{{\mathrm{T}}}{{\left| \hat{Z}(\tau )\right| }^2}}}, \end{aligned}$$

where ${\mathrm{T}}$ is the number of the evaluated signal windows by STFT and which constitutes the first component of the spectrogram feature. Finally, the global description of the spectrogram is encoded with the sum $u^{sp}_{2} = \sum _{\tau =1}^{{\mathrm{T}}}{\hat{Z}(\tau )}$ which is also added to the feature vector. All spectrogram features are then combined to create the two-dimensional spectrogram feature vector ${u^{sp}}^\intercal = [u^{sp}_{1}, u^{sp}_{2}]$.

As a final step, blockwise M-Scan, speckle variance, and the spectrogram features are concatenated in one vector, i.e., $u^\intercal = [{u^{bm}}^\intercal , {u^{bs}}^\intercal , {u^{sv}}, {u^{sp}}^\intercal ]$, which constitutes our final feature vector of length $2N + 3$.

## Experimental results

We now detail the experimental evaluation of our automatic approach compared to manually evaluated M-Scans. We show results on both ex vivo and human clinical data.

**Fig. 4:**
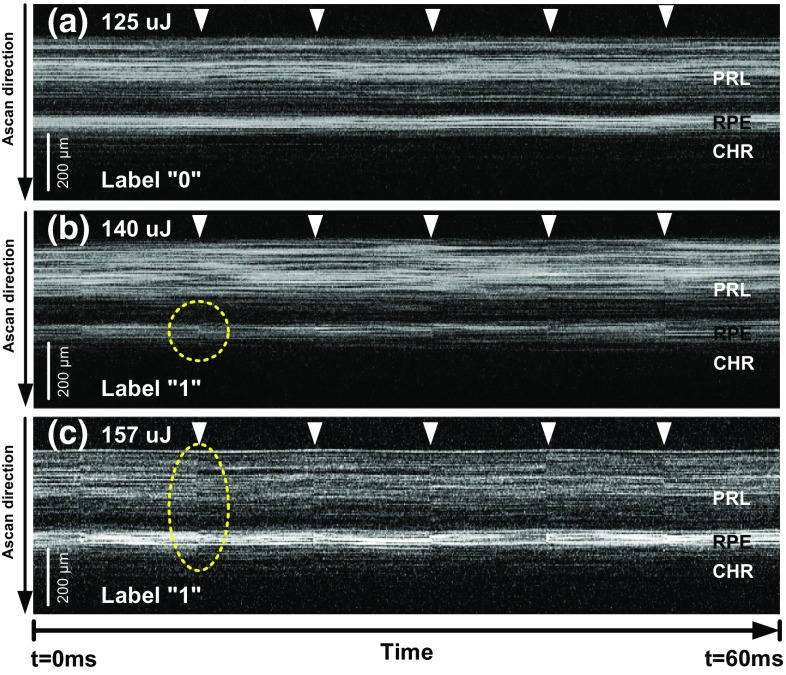
Figure showing 60 ms extracts of examples for manually labeled M-Scans. Figure **a** shows an ex vivo OCT M-Scan with no detectable signal variations labeled as class “0.” **b** and **c** show M-Scans with detectable signal variations limited to the RPE/Bruch’s membrane complex and throughout the retina, respectively. Scans **b** and **c** were consequently labeled as class “1”

### Experimental setup

Two different datasets, namely ex vivo and in vivo human patient data, were collected for the purpose of validation. Enucleated ex vivo porcine eyes were collected from a local slaughterhouse and stored in DMEM solution. M-Scan OCT images were recorded from 153 lesions from 22 enucleated porcine eyes with SRT pulse energies ranging from 110 to 200 $\upmu$J and a retinal treatment spot size of 170 $\upmu$m in order to best represent effects from under- to over-treatment. Scans were acquired at the full speed of 70 kHz to provide the highest possible temporal resolution with incident OCT laser power of 0.7 mW. In addition to the ex vivo data, 16 in vivo human samples were recorded from four patients undergoing clinical SRT. For the treatment, applied pulse energies varied from 40 to 140 $\upmu$J.

Before classification, all OCT M-Scans underwent standard OCT post-processing as described in “System overview” section. To correct for axial motion, the lag of each A-Scan with regard to a reference A-Scan was determined by the calculation of the cross-correlation, and A-Scans were shifted accordingly. The application of the SRT laser was detected using a photodetector sampled at 1 MHz which allowed the determination of the temporal position of laser applications within the M-Scan OCT data stream. Using the information of the photodetector, M-Scans were cropped to the length of the laser pulse train application of 300 ms. This resulted in M-Scans of $600 \times 20000$ pixels.

The ex vivo M-Scans were manually annotated (i.e., successful or unsuccessful treatment) by two independent observers based on the presence of high-frequency signal variations in the M-Scan OCT images as depicted in Fig. 4. Unfortunately, in the case of full eye ex vivo samples, no alternative evaluation method (e.g., histological evaluation) is available, as they suffer from artifacts or require significant preparation of the samples. M-Scans with inconsistent labels (nine out of 162) were excluded from further consideration. From this, the training set consisted of a total of 153 M-Scans from 22 ex vivo eye samples including 119 positive and 34 negative M-Scans. The human in vivo dataset consisted of 16 clinical samples from four different patients. Unlike ex vivo samples, in vivo samples were labeled by an attending ophthalmologist using FFA, from which an assessment regarding each laser application was made and served as groundtruth.

### Classification performance

As described in “M-Scan features” section, blockwise, speckle, and spectrogram features were first extracted from M-Scans. The length of block was set to 353 pixels with a pulse train of 100 Hz and the data acquisition at 70 kHz. The height of the blocks was 300 pixels centered at the RPE. With 30 pulses applied per treatment, this resulted in a total of 60 blocks. For spectrogram features, a Hamming window of length 189 and a hop factor of 9 were experimentally defined. Accordingly, a vector of 123 features was built, and a random forest classifier of 200 trees was built and cross-validated with 50 iterations. In the following, we present the classification performance for ex vivo and in vivo samples.

**Fig. 5:**
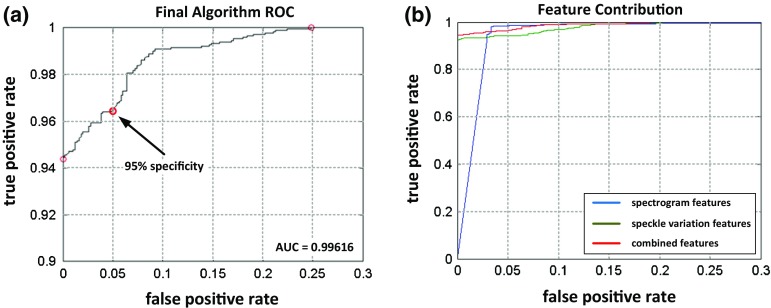
**a** ROC *curve* of our algorithm on ex vivo data. The AUC is 0.996, and the *arrow* indicates the threshold value for 95 % specificity used for clinical evaluation of the classification performance. **b** ROC *curves* showing the performance comparison for different subsets of used features (ex vivo data)

#### Ex vivo performance

The training and testing of the classifier were performed first on ex vivo samples. 102 OCT images out of 153 were used for training, and the rest was used for testing. Fig. 5a depicts the ROC curve of our approach, with an area under curve (AUC) of 0.996. Our results show that a 0.05 false-positive rate (e.g., 1 in 20) still yields over a 0.95 true-positive rate (e.g., 19 in 20) when detecting success treatment applications.

*Feature contribution* In order to assess the impact and importance of the different features used by our classifier, classifiers were retrained using either only spectrogram features or only speckle variation features, as well as all features combined. All other parameters such as numbers of trees, depth, and number of iterations were kept constant. Figure 5b shows the classification performance for these different settings.

As can be seen from these results, the speckle variation features contribute the most to the classification. The spectrogram features alone would already enable reasonable performance but it is only in combination with the others that a high sensitivity and specificity can be attained. This performance may thus be further increased by including phase information and polarization sensitive features.

*Temporal features distribution* One of the ultimate goals of an automatic classification treatment application is the possibility to cease the laser application once the desired effect is attained. In this sense, the temporal distribution of the features is relevant to stopping treatment early. To test the relevance of each part of the M-Scan signal, the algorithm was trained with the features computed identically but with reduced signal length. The M-Scans of 300 ms laser application were divided into three sets of 100 ms (“start,” “middle,” and “end”), and algorithm performance was evaluated for each single set.

**Fig. 6:**
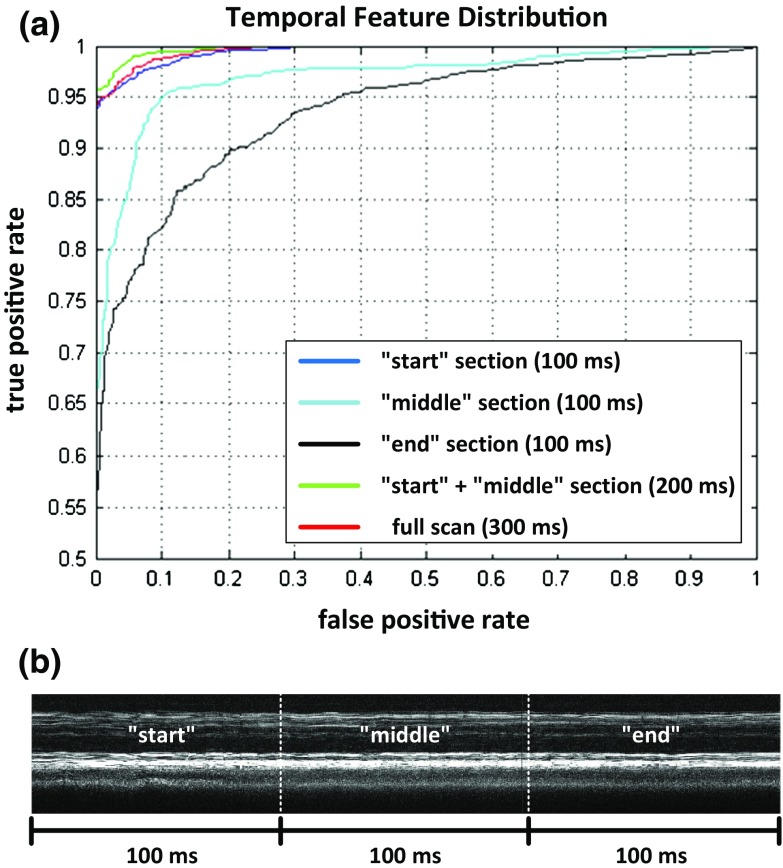
**a** Algorithm performance depending on the duration of laser application and **b** the subdivision of an M-Scan

As can be seen in Fig. 6, the described features are much more prominent within the first two-thirds of the applied pulse train. This may be caused by the breakup of melanin clusters occurring only at the beginning of the pulse application while later pulses merely lead to a rearrangement of the melanin complexes. As the temporal distribution of the features is highly interesting for the extension of the algorithm to a point where it may predict therapy outcome allowing to cease the laser once laser application was successful, future research must focus on the link between the quantitative amount of RPE damage and the extent of OCT signal variations.

Figure 6 shows the results of this analysis and presents the ROC for the each single 100 ms set as well as for the first 200 ms, consisting of the “start” and “middle” set.

#### In vivo performance

In a final step, we trained our algorithm using all the ex vivo M-Scans and keeping the same parameters as in the ex vivo case and then tested our classifier on the in vivo samples which consisted of multiple OCT scans acquired from four patients, two of them having macular edema, one having branch vein occlusion, and a last one with chorioretinopathia centralis serosa. Before laser application, patient pupils were dilated using tropicamid 0.5 % and phenylephrine HCl 2.5 %, and all lesions were applied with pulse energies below the individual ophthalmoscopic visibility threshold. The classification results were compared to both FFA images and manual OCT labeling. Table 1 shows the results on the 16 clinical SRT applications with FFA leakage evaluated by the attending ophthalmologist. The algorithm performance was evaluated at threshold values for 100 % specificity, 95 % specificity, and 100 % sensitivity. The best performance was thereby achieved using threshold for 95 % specificity leading to a correct prediction of SRT outcome in 15 out of 16 cases corresponding to a success rate of 93.8 % when compared to the FFA analysis.

**Table 1:** Performance analysis of the classification algorithm on clinical in vivo SRT data

Laser energy [$\upmu$J]	FFA label	OCT label	Classification 100 % specificity	Classification 95 % specificity	Classification 100 % sensitivity
120	1	1	1	1	1
120	1	1	1	1	1
60	0	0	0	0	*1*
80	0	0	0	0	*1*
80	0	*1*	*1*	*1*	*1*
80	–	1	1	1	1
80	1	1	1	1	1
80	1	*0*	*0*	1	1
80	1	1	*0*	1	1
120	1	1	1	1	1
60	1	1	1	1	1
80	1	1	1	1	1
100	1	1	1	1	1
100	1	1	1	1	1
100	1	1	1	1	1
100	1	1	1	1	1
Accuracy (%)		87.5	81.3	93.8	81.3

Classifications were evaluated for 100 and 95 % specificity as well as for 100 % sensitivity. Classification results and the manual labeling were compared to the FFA visibility as assessed by the attending ophthalmologist. Classification performance was best for 95 % specificity, and false results are highlighted in italics

With the classification tuned to prevent false-positive results, the algorithm classified one lesion as positive where no leakage was detected in the FFA. However, visual inspection of the OCT scan revealed the presence of the described features; it thus remains unclear whether the result is really a false-positive classification or not. For this corresponding lesion, pathological features in the area of treatment may have covered or omitted visible leakage in the FFA. There is, unfortunately, no way to further determine the true outcome of the treatment. The result was thus kept as a false classification.

Despite the fact that the algorithm in this paper was trained exclusively on ex vivo data, its performance on in vivo human data is remarkably high. This can be somewhat explained by the fact that SRT omits heat dissipation and convection at too short timescales so that cooling effects due to blood perfusion do not play any role in the assessment. Nevertheless, this is a significant outcome as further studies may be based on enucleated porcine eyes which include no ethical considerations and are widely available. Moreover, we observed that the algorithm correctly classified also one lesion as a positive RPE rupture which was in agreement with the FFA while the visual inspection of the OCT data revealed no features. This finding may be a sign of the fact that the algorithm shows a higher specificity than visual inspection by human annotators.

## Conclusion

In this manuscript, we have introduced a novel strategy for automatic classification of SRT lesions using M-Scan OCT data. The presented features and algorithm showed encouraging performance and provided classification results in good agreement with clinical evaluation of lesions by FFA. In particular, our approach provided correct prediction of RPE leakage in 15 out of 16 cases in a preliminary human clinical trial. The proposed OCT-SRT system associated with a classification approach showed good performance for ex vivo samples, and we demonstrated that our approach can be trained using ex vivo data even when evaluating in vivo data. In addition, the performance of the algorithm was found to be independent of the lateral position of the measurement spot within the treatment area as long as the measurement and treatment area are fully overlapped.

Such OCT-based feedback and lesion classification may be extended to a predictive classification which can be used to cease the laser once the desired effect is achieved and may serve as an additional safety net for sub-threshold therapies. Moreover, with the inclusion of volumetric data, captured before and after laser application, the algorithm performance may further be improved, enabling a more detailed classification. The use of OCT data has the potential of providing treatment feedback which is crucial for a reliable and repeatable therapy and would eventually support the proliferation of the reliable and cost-effective SRT as a treatment option.
